# Inflammatory response in the mid colon of ICR mice treated with polystyrene microplastics for two weeks

**DOI:** 10.1186/s42826-021-00109-w

**Published:** 2021-11-22

**Authors:** Yun Ju Choi, Ji Eun Kim, Su Jin Lee, Jeong Eun Gong, You Jeong Jin, Sungbaek Seo, Jae Ho Lee, Dae Youn Hwang

**Affiliations:** Department of Biomaterials Science (BK21 FOUR Program), College of Natural Resources & Life Science/Life and Industry Convergence Research Institute/Laboratory Animals Resources Center, Pusan National University, Miryang, South Korea

**Keywords:** Microplastics, Inflammation, Colon, NF-κB, Cytokines

## Abstract

**Background:**

The oral administration of polystyrene-microplastics (PS-MPs) causes chronic constipation of ICR mice, but there are no reports on their effects on the inflammatory response in the colon. To determine if the oral administration of MPs causes inflammation in the colon, the changes in the apoptosis-associated speck like protein containing a caspase recruitment domain (ASC)-inflammasome pathway, nuclear factor kappa-light-chain-enhancer of activated B cells (NF-κB) signaling pathway, and inflammatory cytokine expression were evaluated in the mid colon of ICR mice treated with 0.5 μm size PS-MPs for two weeks.

**Results:**

The thicknesses of the mucosa, muscle, flat luminal surface, and crypt layer were decreased significantly (*p* < 0.01) in the mid colon of the MPs treated group compared to the Vehicle treated group. On the other hand, a remarkable increase in the expression levels of NOD-like receptor pyrin domain-containing protein (NLRP) 3, ASC, and Cleaved Caspase (Cas)-1 protein was observed in the MPs treated group. In addition, similar increasing pattern in the levels of p-NF-κB and phospho-inhibitory subunit of NF-κB (p-IkB) α protein was detected. Four inflammatory cytokines, including NF-κB, interleukin (IL)-6, tumor necrosis factor (TNF)-α, and IL-1β, showed an increased expression level after the MPs treatment.

**Conclusions:**

Therefore, the present study suggests that PS-MPs can be a novel cause of an inflammatory response in the mid colon of ICR mice.

## Background

Inflammation plays an important role in the complex biological response of the host defenses to various harmful stimuli, including infectious agents, tissue injury, and irritants [1]. Among these stimuli, pathogens, allergens, toxins, and frostbite are causes of acute inflammation, whereas smoking, obesity, poor diet, and stress promote chronic inflammation [2, 3]. The above stimulators induce the activation of inflammatory cells and the regulation of inflammatory mediators by triggering the inflammatory signaling pathways, including the NF-κB, mitogen-activated protein kinase (MAPK), and Janus kinase (JAK)-signal transducer and activator of transcription (STAT) pathway [4]. During this process, tissues are accompanied by several cardinal signs, such as heat, pain, redness, swelling, and loss of function to eliminate the initial cause of damage, protects the undamaged tissue, and initiates tissue repair [5]. Furthermore, inflammation is a common pathological mechanism underlying various chronic diseases: cancer, diabetes, cardiovascular disease, inflammatory bowel disease (IBD), and arthritis [6]. Moreover, inflammasome has been understood as one of molecular mechanism for inflammation. It is an intracellular complex receptor and sensor that response to broad ranges of pathogenic microorganisms and molecules derived from host proteins through the activation of Cas-1 and induction of inflammation [7, 8]. During response of the innate immunity, pattern recognition receptors (PRRs) and damage-associated molecular patterns (DAMPs) are sensed by inflammasome, and then their multimeric protein complex assembles as mature form that contain a nucleotide-binding oligomerization domain-like receptor (NLR) protein as the main component [9, 10]. Also, this includes the following four types; the NLRP3/NALP3 inflammasome [11], the NLRC4/IPAF inflammasome [12, 13], the NLRP1/NALP1b inflammasome [14], and the AIM2 (absent in melanoma 2) containing inflammasome [15, 16]. However, inflammasomes have not received great attention for the inflammatory response of MPs until recently, although alternative regulation of some cytokines was analyzed after MPs treatment [17].

There are conflict results on whether MPs are a cause of the inflammatory response in vitro and in vivo. Some studies show that treatment with MPs induces an inflammatory response in cells and several tissues of mice. In A549 alveolar basal epithelial cells, the mRNA transcription levels of proinflammatory cytokines, including IL-6, IL-8, and TNF-α, were upregulated significantly (*p* < 0.05) after treatment with 70 nm polystyrene-nanoparticles (PS-NPs) at 160 μg/mL; 25 nm and 70 nm PS-NPs did not induce a change in the IL-1β transcription levels [18]. Furthermore, PS-NPs (4.06 ± 0.44 µm) treatment induced the protein expression of proinflammatory cytokines, including IL-6 and IL-8, in BEAS-2B cells [19], while ~ 20 µm PS particles stimulate the release of some cytokines in RAW264.7 cells [20]. Carboxylated PS-NPs induced a similar response. IL-8 cytokine secretion in monocytes was increased after a 20 nm PS-NPs treatment, but IL-6 and IL-8 secretion from monocytes and macrophages was stimulated by a 500 and 1,000 nm PS-NPs treatment [21]. A similar response was detected in a mouse model. In C57BL/6 mice treated with polyethylene (PE)-MPs at 6, 60, and 600 μg/day for five consecutive weeks, the serum level of IL-1α, IL-6, IL-9, and regulated on activation normal T cell expressed and secreted (RANTES) were increased significantly (*p* < 0.05). At the same time, the chronic inflammatory cells, including lymphocytes and plasma cells, were infiltrated into the lamina propria of the 600 μg/day treated mice group [22]. On the other hand, other studies have shown that repeated-dose exposure of MPs cannot induce any significant (*p* < 0.05) inflammatory response. A treatment of C57BL/6NTac mice with 1, 4, and 10 μm PS-MPs did not affect β-galactosidase expression derived from the inflammation-sensitive heme oxygenase 1 promoter and histologically detectable lesions in the colon, even though a minor fraction was successfully uptaken into the cells [23].

This study examined whether PS-MPs administration can cause an inflammatory response in the mid colon of mice through an analysis of the histopathology, inflammasome proteins expression, NF-κB signaling pathway activation, and cytokines expression. The results suggest that PS-MPs administration is a novel cause of the inflammatory response in the mid colon of ICR mice.

## Results

### Effects of MPs administration on the histopathological structure of the mid colon of ICR mice

**Fig. 1:**
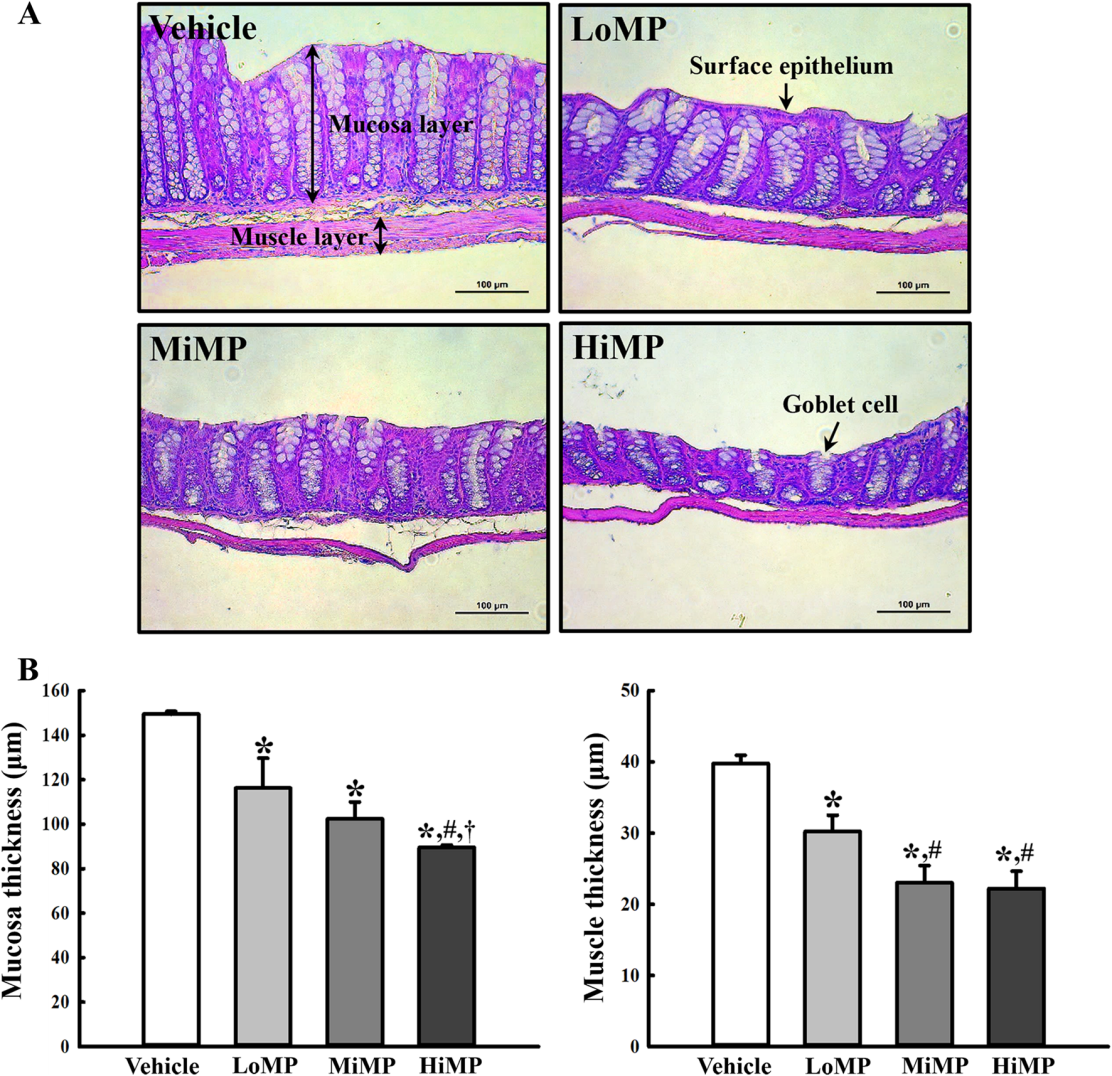
Histopathological structures of the mid colon. **A** H&E stained sections of the mid colon from the Vehicle, LoMP, MiMP, and HiMP treated groups were observed at 200 × magnification using an optical microscope. **B** The histopathological parameters were determined using the Leica Application Suite. Four to six mice per group were used to prepare the H&E stained slides, and the histopathological parameters were measured in duplicate for each slide. The data are reported as the mean ± SD. *p < 0.05 compared with Vehicle treated group; #*p* < 0.05 compared with LoMP treated group; **†***p* < 0.05 compared with MiMP treated group. Abbreviation: LoMP, Low concentration of microplastics; MiMP, Medium concentration of microplastics; HiMP, High concentration of microplastics

This study examined whether MPs administration could affect the histopathological structure of mid colon in ICR mice by measuring the changes in the H&E-stained histopathological structures in the mid colon of the subset groups. Significant (*p* < 0.01) changes in the thicknesses of the mucosa, muscle, and surface epithelium were observed in the three MPs treated groups compared to the Vehicle group. These levels were lower in the MPs treated group than the Vehicle treated group. In addition, the number of goblet cells was lower in the MPs treated group than the Vehicle treated group (Fig. 1). These results suggest that MPs administration is associated with abnormalities in the histopathological structure of the mid colon in ICR mice.

### Effects of MPs administration on the ASC-inflammasome pathway

**Fig. 2:**
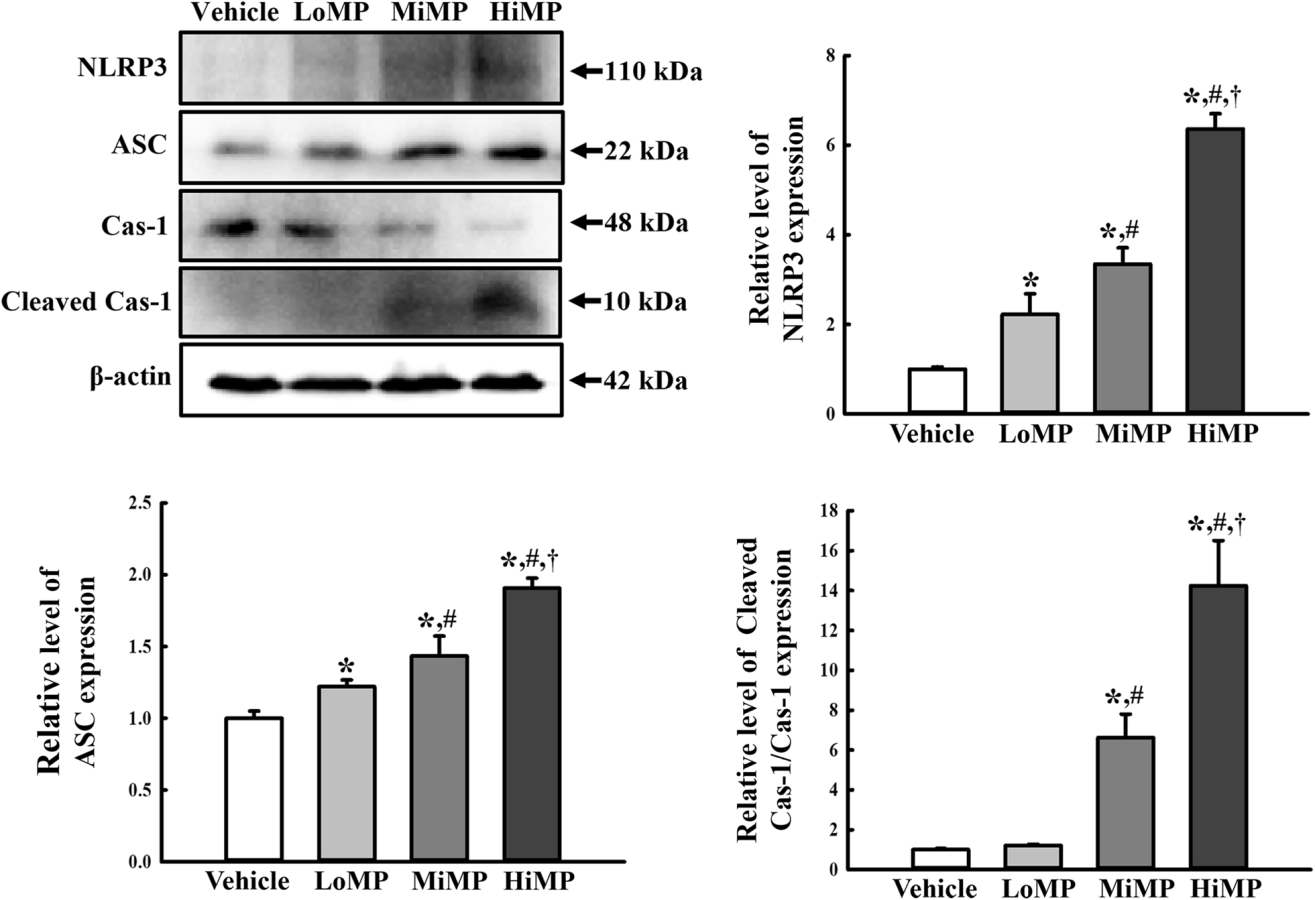
Expressions of the key mediators in the ASC-inflammasome signaling pathway. Expression levels of NLRP3, ASC, Cas-1, and Cleaved Cas-1 in the ASC-inflammasome signaling pathway were measured by Western blot analysis using the specific primary antibodies and HRP-labeled anti-rabbit IgG antibody. After the intensity of each band was determined using an imaging densitometer, the relative levels of the four proteins were calculated based on the intensity of actin. Four to six mice per group were used to prepare the total tissue homogenate, and Western blot analyses were assayed in duplicate for each sample. Data are reported as the mean ± SD. *p < 0.05 compared with Vehicle treated group; **#***p* < 0.05 compared with LoMP treated group; **†***p* < 0.05 compared with MiMP treated group. LoMP, Low concentration of microplastics; MiMP, Medium concentration of microplastics; HiMP, High concentration of microplastics; NLRP3, NOD-like receptor pyrin domain-containing protein 3; ASC, Apoptosis-associated speck-like protein containing a C-terminal caspase recruitment domain

The altered expression levels of NLRP3, Cleaved Cas-1/Cas-1, and ASC were measured in the mid colon of MPs treated ICR mice to determine if the histological abnormality of the mid colon is linked to the ASC-inflammasome pathway. First, we analyzed the expression level of NLRP3 because it has been considered as a critical component in response to infections of microorganisms including fungi, bacteria and virus, and cellular damages. The level of NLRP3 expression increased dose-dependently in the MPs treated group compared to the Vehicle treated group (Fig. 2a and b). A similar pattern was detected in the expression level of the ASC proteins and Cleavage Cas-1/Cas-1 proteins (Fig. 2a, c, and d). In particular, the total level of Cas-1 protein decreased significantly (*p* < 0.01) in a dose-dependent manner in the MPs treated group (Fig. 2a and d). These results suggest that histological abnormality of the mid colon is associated with upregulation of the ASC-inflammasome pathway in MPs treated ICR mice.

### Effects of MPs administration on the NF-κB signaling pathway

**Fig. 3:**
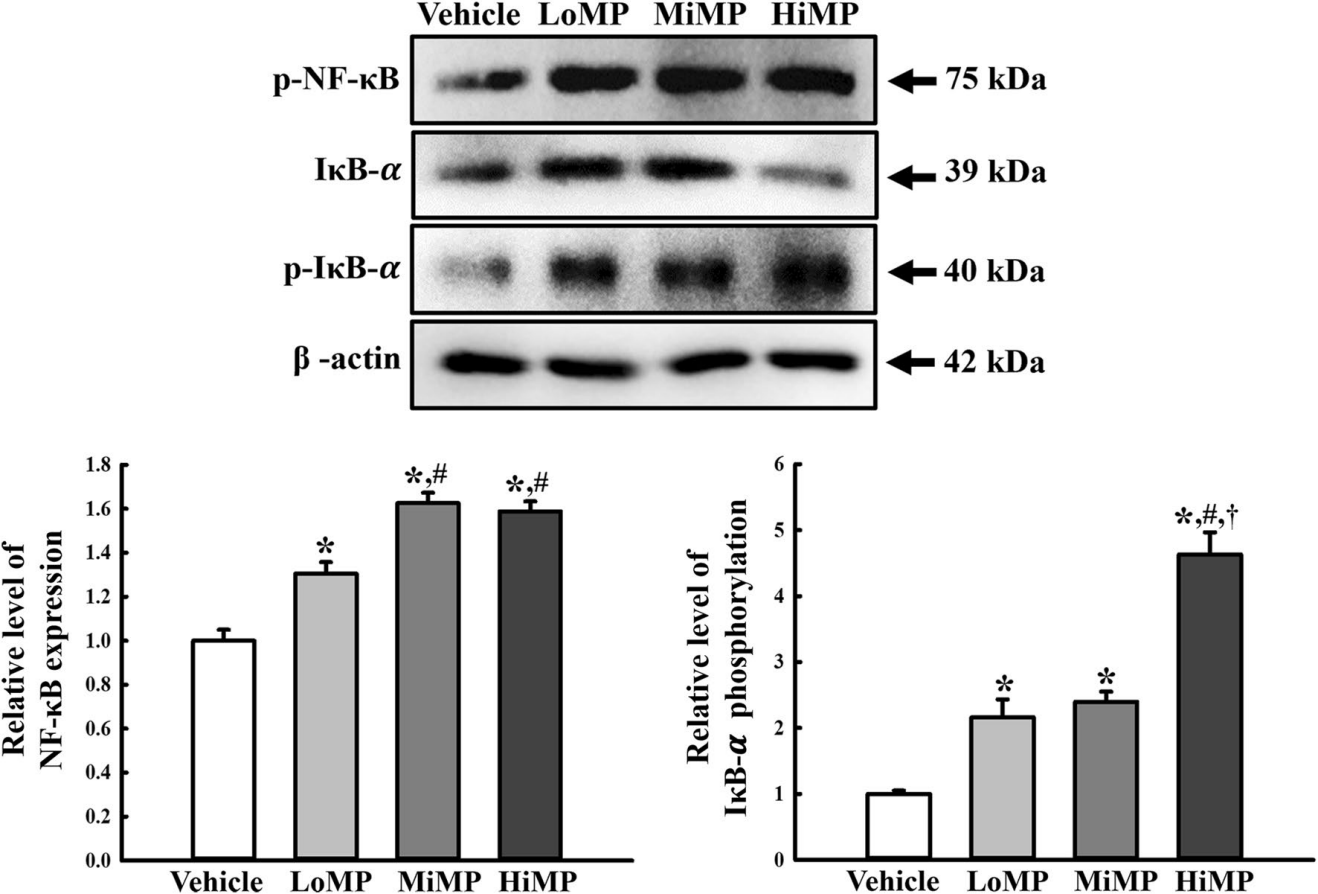
Expressions of key mediators in NF-κB signaling pathway. Expression levels of p-NF-κB, p-IκB-α, and IκB-α in the NF-κB signaling pathway were measured by Western blot analysis using the specific primary antibodies and HRP-labeled anti-rabbit IgG antibody. After the intensity of each band was determined using an imaging densitometer, the relative levels of the three proteins were calculated based on the intensity of actin. Four to six mice per group were used to prepare the total tissue homogenate, and Western blot analyses were assayed in duplicate for each sample. Data are reported as the mean ± SD. *p < 0.05 compared with Vehicle treated group; **#***p* < 0.05 compared with LoMP treated group; **†***p* < 0.05 compared with MiMP treated group. LoMP, Low concentration of microplastics; MiMP, Medium concentration of microplastics; HiMP, High concentration of microplastics; NF-κB, Nuclear factor κB

This study examined whether upregulation of the ASC-inflammasome pathway is accompanied by changes in the NF-κB signaling pathway during a histological abnormality induced by MPs administration. The altered phosphorylation levels of NF-κB and IκB were measured in the mid colon of MPs treated ICR mice. The level of the p-NF-κB protein was enhanced remarkably in the mid colon of the MPs treated ICR mice, even though the highest level was observed in the HiMP treated group. A similar alteration pattern in the level of p-IκB-α was also detected in the same group (Fig. 3). These results suggest that the upregulation of the ASC-inflammasome pathway may be associated with the activation of the NF-κB signaling pathway during a histological abnormality induced by MPs administration.

### Effects of MPs administration on the inflammatory and anti-inflammatory cytokines

**Fig. 4:**
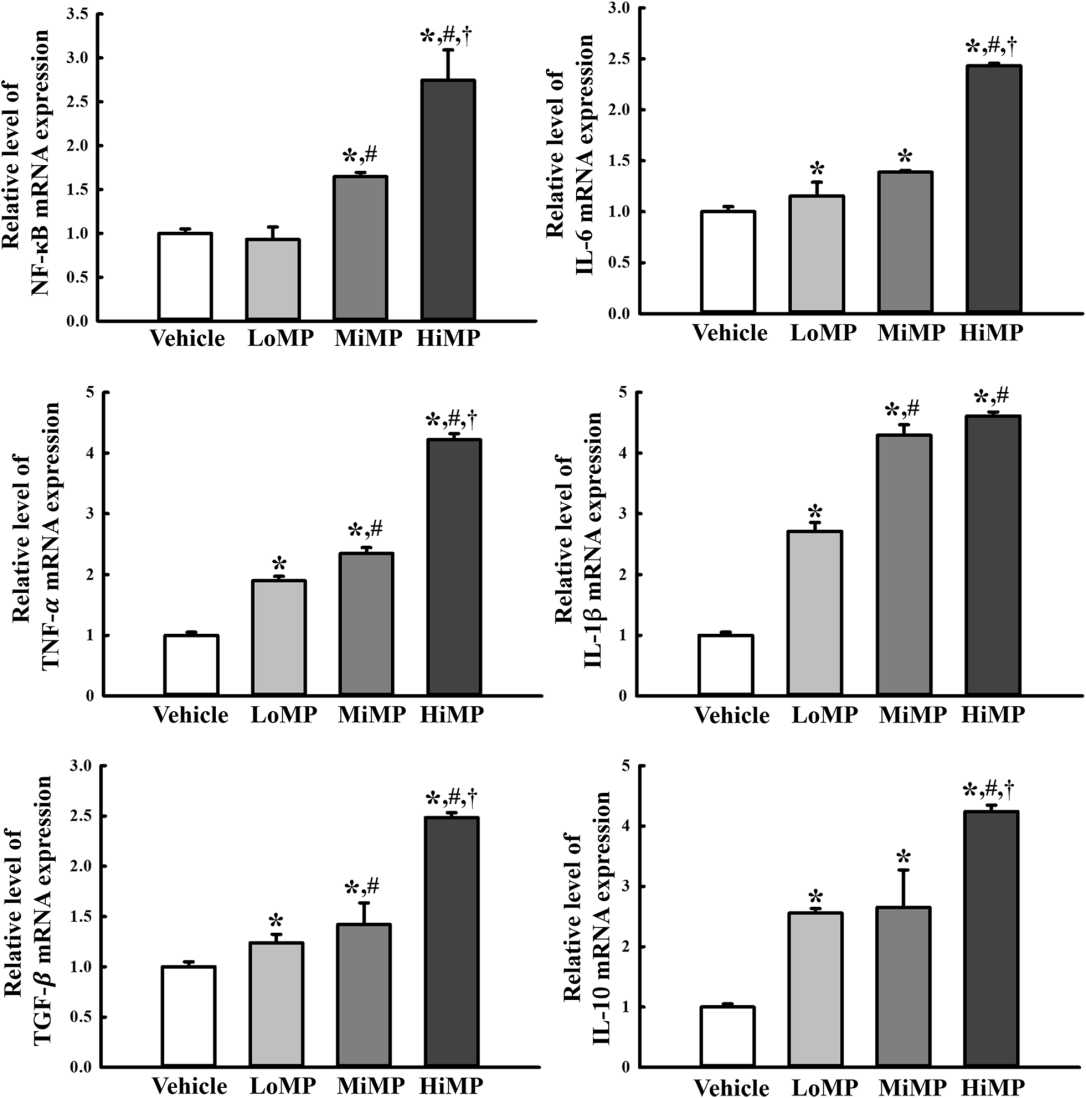
Transcription of inflammatory cytokines. The levels of NF-κB, IL-6, TNF-α, IL-1β, TGF-β, and IL-10 transcripts in the total mRNA of mid colons were measured by RT-qPCR using specific primers. The mRNA levels of the six genes were calculated based on the intensity of actin as an endogenous control. Four to six mice per group were used to prepare total RNA; RT-qPCR analyses were assayed in duplicate for each sample. The data are reported as the mean ± SD. *p < 0.05 compared with Vehicle treated group; **#***p* < 0.05 compared with LoMP treated group; **†***p* < 0.05 compared with MiMP treated group. LoMP, Low concentration of microplastics; MiMP, Medium concentration of microplastics; HiMP, High concentration of microplastics; RT-qPCR, Quantitative real time-PCR; NF-κB, Nuclear factor kappa-light-chain-enhancer of activated B cells; IL-6, Interleukin-6; TNF-α, Tumor Necrosis Factor-α; IL-1β, Interleukin-1β; TGF-β, Transforming Growth Factor-β

Finally, this study examined whether activation of the NF-κB signaling pathway is accompanied by the upregulation of inflammatory cytokines. To achieve this, the transcription levels of NF-κB, TNF-α, IL-6, IL-1 $\beta$, IL-10, and TGF-β1 were evaluated in the mid colon in the MPs treated ICR mice. The mRNA levels of NF-κB, TNF-α, IL-6, IL-1 $\beta$, IL-10, and TGF-β1 cytokines were increased significantly (*p* < 0.01) in the mid colon of the MPs treated group compared to the Vehicle treated group (Fig. 4). Therefore, activation of the NF-κB signaling pathway is associated with the regulation of the inflammatory and anti-inflammatory cytokines in the mid colon of ICR mice treated with MPs for two weeks.

## Discussion

A MPs treatment caused various physiological responses, including modification of the gut microbiota composition, decrease in mucus secretion and increase in inflammatory cytokines in the intestine [22, 24, 25]. In particular, the oral administration of PS-MPs into ICR mice induces chronic constipation that is characterized by the dysregulation of gastrointestinal (GI) motility, mucin secretion, and chloride ion and water transportation in the mid colon [26]. As part of a study on the correlation between MPs and constipation, this study examined the inflammatory response in the mid colon during MPs induced constipation. The results showed significant (*p* < 0.01) alterations on the ASC-inflammasome pathway, NF-κB signaling pathway, and inflammatory cytokine expression induced in the mid colon of ICR mice treated with MPs for two weeks. These data provide the first evidence that MPs induced constipation may be associated with an inflammatory response.

The NF-κB signaling pathway plays an important role in the inflammatory response, host immune response, the proliferation and survival of cells, and apoptosis [27]. When the cells have received various stimuli, including pathogen-derived substances, intracellular cytokines, and many enzymes, activated IκB kinase (IKK) promotes the phosphorylation of IκB. Subsequently, NF-κB is released and is translocated to the nucleus to stimulate the transcription of inflammatory cytokines [28, 29]. Actually, these signaling pathway was strongly influenced by the PS-MPs treatment. The expression levels of four inflammation-related genes (TRPV1, iNOS, IL-1β, and IL-8) were decreased significantly (*p* < 0.05) in the human colorectal adenocarcinoma Caco-2 cells treated with three different concentrations (12.5, 25, and 50 mg/L) of PS-MPs for 24 and 48 h [30]. Our study examined the activation of NK-κB signaling and expression of inflammatory cytokines in the colon of ICR mice treated with three different concentrations (10, 50, and 100 μg/mL) of PS-MPs for two weeks. Significant (*p* < 0.01) enhancement of four cytokines (NF-κB, IL-6, TNF-α, and IL-1β) was detected after the PS-MPs treatment through activation of NF-κB. The findings of the present study were different from those of a previous study that showed a decrease in inflammatory gene expression in MPs treated Caco-2 cells. This difference was attributed to the difference in experimental conditions of cells and animals, even though further research will be needed to verify the molecular mechanism.

Inflammasomes form mainly from five members of pattern-recognition receptors (PRRs): the nucleotide-binding oligomerization domain (NOD), leucine-rich repeat (LRR)-containing proteins (NLR) family members NLRP1, NLRP3, and NLR Family CARD domain containing (NLRC) 4, as well as absent-in-melanoma 2 (AIM2) and pyrin [31, 32]. They are a critical intracellular component for the host innate immune system against a broad range of microorganisms, including bacterial, fungal, and viral infections, by regulating Cas-1 activation and proinflammatory cytokine secretion [7, 33]. During the innate immune response, inflammasome is recognized as pathogen-associated molecular patterns (PAMPs) or damage-associated molecular patterns (DAMPs) to eliminate microbial pathogens and repair damaged tissues [9]. Inflammasome is activated by various extracellular stimuli, including toxin, ATP, RNA virus, and particular matter, as well as diverse cellular events, such as ionic influx, mitochondrial dysfunction, reactive oxygen species (ROS) production, and some lysosomal damage [34]. Furthermore, the aberrant activation of the inflammasome is tightly linked with several inflammatory diseases, which involve diabetes, alzheimer’s disease, atherosclerosis, and cryopyrin-associated periodic [34]. In this study, the ASC-inflammasome pathway was analyzed to examine the effects of MPs administration on inflammatory response in the mid colon of mice. The levels of NLRP3, ASC, and Cleaved Cas-1 proteins were increased in a dose-dependent manner in the MPs treated group, as shown in Fig. 3. These results provided the first evidence that the oral administration of MPs may be associated with the activation of inflammasome in the mid colon of ICR mice. Further research will be needed to determine which external stimulus or intracellular events contribute to inflammasome activation induced by MPs.

## Conclusions

This study investigated newly characterized inflammatory response in the mid colon of ICR mice orally administrated MPs for two weeks. These data provide novel evidence that the oral administration of MPs is tightly correlated with the activation of the ASC-inflammasome pathway, activation of the NF-κB signaling pathway, and the upregulation of inflammatory cytokines expression. Therefore, MPs is considered as one of the novel causes of inflammation in the colon of ICR mice.

## Methods

### Experimental design of the animal study

The animal protocol to characterize the constipation phenotype was reviewed and approved by the Pusan National University-Institutional Animal Care and Use Committee (PNU-IACUC) based on the ethical procedures and scientific care (Approval Number PNU-2020–2654). All ICR mice were maintained at the Pusan National University-Laboratory Animal Resources Center, accredited by the Korea Food and Drug Administration (KFDA) (Accredited Unit Number-000231) and the Association for Assessment and Accreditation of Laboratory Animal Care (AAALAC) International (Accredited Unit Number; 001525). All mice were provided access to a standard irradiated chow diet (Samtako BioKorea Inc., Osan, Korea) and water ad libitum. Throughout the experiment, the mice were maintained in a specific pathogen-free (SPF) state under a strict light cycle (on at 08:00 h; off at 20:00 h) at 23 ± 2°C and 50 ± 10% relative humidity.

Briefly, seven-week-old ICR mice (n = 24) were assigned to a 1× PBS treated group (Vehicle, n = 6) or MPs treated group (MP, n = 18). The MPs treated group was subdivided into a low concentration MPs treated group (LoMP, n = 6), medium concentration MPs treated group (MiMP, n = 6), and high concentration MPs treated group (HiMP, n = 6). The optimal dosage for MP administration used in the animal model was chosen based on previous studies for tissue accumulation [35], and effects on gut homeostasis [24]. The three MPs treated groups were administrated orally a dispersed MPs solution of varying concentrations (10 μg/mL, 50 μg/mL and 100 μg/mL) once daily (0.5 mL/day) for two weeks, while the Vehicle treated group was administered the same volume of 1× PBS solution. The MPs had a particle size of 0.5 μm and a density of 1.04–1.06 g/cm^3^ in aqueous suspensions at 25 mg/mL (Sigma-Aldrich Co., St. Louis, MO, USA). The physiological condition of all mice in each group was monitored regularly at 10 a.m. every day during the experimental periods; there were no occurrences of severely ill or dead animals. Two weeks after MPs administration, all mice were euthanized using CO_2_ gas, after which the mid colon tissue was acquired and stored at -70°C in Eppendorf tubes until assayed.

### Histopathological analysis of mid colon

The mid colons collected from the Vehicle, LoMP, MiMP, and HiMP groups were fixed in 10% formalin for 48 h. The tissue samples were then embedded in paraffin wax, after which they were cut into 4 μm thick sections and stained with hematoxylin and eosin (H&E, Sigma-Aldrich Co.). The sections were then analyzed by optical microscopy to determine the mucosal thickness, muscle thickness, surface epithelium thickness, and number of goblet cells in the mid colons, applying the Leica Application Suite (Leica Microsystems Ltd., Heerbrugg, Switzerland).

### Western blotting analysis

The Pro-Prep Protein Extraction Solution (Intron Biotechnology Inc., Seongnam, Korea) was used to prepare the total proteins from mid colons of the Vehicle, LoMP, MiMP, and HiMP groups, according to the manufacturer’s protocol. Protein homogenates were then centrifuged at 13,000 rpm at 4°C for 5 min, after which total protein concentrations were determined using a SMARTTM Bicinchoninic Acid Protein assay kit (Thermo Fisher Scientific Inc., Wilmington, MA, USA). The total proteins (30 μg) were subjected to 4–20% sodium dodecyl sulfate–polyacrylamide gel electrophoresis (SDS-PAGE) for 3 h, and the resolved proteins were transferred to nitrocellulose membranes for 2 h at 40 V. The membranes were then probed overnight with the following primary antibodies at 4°C: anti-NLRP3 (Cell Signaling Technology Inc., Cambridge, MA, USA.), anti-ASC (Cell Signaling Technology Inc.), anti-Cas-1 (Cell Signaling Technology Inc.), or anti-β-actin (Sigma-Aldrich Co.). The membranes were then washed with a washing buffer (137 mM NaCl, 2.7 mM KCl, 10 mM Na_2_HPO_4_, 2 mM KH_2_PO_4_, and 0.05% Tween 20), followed by incubation with 1:1,000 diluted horseradish peroxidase-conjugated goat anti-rabbit IgG (Zymed Laboratories, South San Francisco, CA, USA) for 2 h at room temperature. Subsequently, the blots were developed using a Chemiluminescence Reagent Plus kit (Pfizer Inc., Gladstone, NJ, USA). Signal images of each protein were then acquired using a digital camera (1.92 MPs resolution) of the FluorChem® FC2 Imaging system (Alpha Innotech Corporation, San Leandro, CA, USA). The protein densities were semi-quantified using the AlphaView Program, version 3.2.2 (Cell Biosciences Inc., Santa Clara, CA, USA).

### Quantitative realtime – polymerase chain reaction (RT-qPCR) analysis

Frozen mid colon tissue was chopped with scissors and homogenized in a RNA Bee solution (Tet-Test, Friendswood, TX, USA). The total RNA molecules were isolated by centrifugation at 15,000 rpm for 15 min, after which the RNA concentration was measured using a NanoDrop Spectrophotometer (Allsheng, Hangzhou, China). Approximately 5 µg of the total RNA was annealed with 500 ng of oligo-dT primer (Thermo Fisher Scientific Inc.) at 70°C for 10 min. Complementary DNA (cDNA) was synthesized using the Invitrogen Superscript II reverse transcriptase (Thermo Fisher Scientific Inc.). qPCR was performed with the cDNA template obtained (2 µL) and 2 × Power SYBR Green (6 µL; Toyobo Life Science, Osaka, Japan) containing the following specific primers: NF-κB sense primer 5’-AAGAC AGAAA TAAGG AAGGG TGGTA A-3’ and antisense primer 5’-TGACC TCACT GCTAA ACTCT GAACA-3’; IL-6 sense primer 5’-TTGGG ACTGA TGTTG TTGAC A-3’, antisense primer 5’-TCATC GCTGT TGATA CAATC AGA-3’; TNF-$\alpha$ sense primer 5’-CCTGT AGCCC ACGTC GTAGC-3’, antisense primer 5’-TTGAC CTCAG CGCTG ACTTG-3’; IL-1 $\beta$ sense primer 5’-CTACA GGCTC CGAGA TGAAC AAC-3’ and antisense primer 5’-TCCAT TGAGG TGGAG AGCTT TC-3’; transforming growth factor (TGF)-$\beta$ 1, sense primer 5’-GAGGT CACCC GCGTG CTA-3’, antisense primer 5’-TGTGT GAGAT GTCTT TGGTT TTCTC-3’; IL-10, sense primer 5’-CAGCC GGGAA GACAA TAACT G-3’, antisense primer 5’-CCGCA GCTCT AGGAG CATGT-3’; β-actin sense primers 5’-ACGGC CAGGT CATCA CTATT G-3’ and antisense primer 5’-CAAGA AGGAA GGCTG GAAAA GA-3’, respectively. qPCR was performed for 40 cycles using the following sequence: denaturation at 95°C for 15 s, followed by annealing and extension at 70°C for 60 s. The fluorescence intensity was measured at the end of the extension phase of each cycle. The threshold value for the fluorescence intensities of all samples was set manually. The reaction cycle at which the PCR products exceeded this fluorescence intensity threshold during the exponential phase of PCR amplification was considered the threshold cycle (Ct). Expression of the target gene was quantified relative to the housekeeping gene β-actin, based on a comparison of the Cts at a constant fluorescence intensity according to Livak and Schmittgen’s method [36].

### Statistical analysis

Statistical significance was evaluated using the One-way Analysis of Variance (ANOVA) (SPSS for Windows, Release 10.10, Standard Version, Chicago, IL, USA), followed by a Tukey post hoc t-test for multiple comparisons. All values are expressed as the means ± SD. A p-value *p* < 0.05 was considered significant.

## Data Availability

All the data that support the findings of this study are available on request from the corresponding author.

## Abbreviations

ASC: Apoptosis-associated speck-like protein containing a C-terminal caspase recruitment domain;
NF-κB: Nuclear factor kappa-light-chain-enhancer of activated B cells
NLRP3: NOD-like receptor pyrin domain-containing protein 3
PNU -IACUC: Pusan national university-laboratory animal care and use committee
PS-MPs: Polystyrene-microplastics
TGF-β: Transforming growth factor-β
TNF-α: Tumor necrosis factor-α
